# The Relationship Between Intolerance of Uncertainty and Employment Anxiety of Graduates During COVID-19: The Moderating Role of Career Planning

**DOI:** 10.3389/fpsyg.2021.694785

**Published:** 2021-10-26

**Authors:** Li Chen, Shuyu Zeng

**Affiliations:** School of Psychology, Northwest Normal University, Lanzhou, China

**Keywords:** COVID-19, purpose, career planning, intolerance of uncertainty, employment anxiety, graduates

## Abstract

COVID-19, which is characterized by uncertainty, makes the employment anxiety of college graduates in the period of career change more and more intense. How to deal with this challenge is particularly important. The present study took career planning as a moderating variable to explore the relationship between intolerance of uncertainty (IU) and employment anxiety, as well as the role of career planning. In this quantitative study, the data of 563 college graduates from different schools were collected by using the Intolerance of Uncertainty Scale-12, Vocational Selection Anxiety Questionnaire of University Graduate, and Career Planning Scale. The final effective sample size was 550 (the overall recovery rate was 97.7%). The results of correlation analysis showed that there was a significant positive correlation between IU and employment anxiety, while there was a significant negative correlation between career planning and IU, as well as the relationship between career planning and employment anxiety. The results of hierarchical regression analysis showed that IU significantly positively predicted the employment anxiety of graduates, and career planning moderated the relationship between IU and employment anxiety. These findings suggested that maintaining a sense of career planning can help college graduates get through smoothly in the face of uncertainty of COVID-19.

## Introduction

Existing literature has discussed the relationship between intolerance of uncertainty and employment anxiety (Li et al., 2012), as well as the uncertainty caused by COVID-19 and its impact on career development (Akkermans et al., 2020; Blustein and Guarino, 2020). However, there is a lack of academic research on the importance of career planning in relation to a sense of purpose. The purpose of the present study was to strengthen the exploration of the moderating role of career planning between intolerance of uncertainty and employment anxiety in the context of COVID-19, in order to determine the impact on university graduates.

Since the end of 2019, coronavirus disease 2019 (COVID-19), a novel disease caused by severe acute respiratory syndrome coronavirus 2 (SARS-Cov-2) has spread rapidly around the world. On January 30, 2020, the World Health Organization announced that the coronavirus disease 2019 was a public health emergency of international concern (World Health Organization, 2020). There is no doubt that COVID-19 brings about a deep uncertainty, which is obvious in interpersonal relationships, work, our inner life, and our means of survival (Blustein and Guarino, 2020). Among them, the sharp rise in unemployment rate and the consequent decrease in sources of support and job opportunities have become the main characteristics of work (Blustein and Guarino, 2020). This is a major shock to many people’s careers (Akkermans et al., 2020). Especially for college graduates facing a special development period, the development of this emerging adulthood is usually related to worry, pressure, and uncertainty (Schulenberg et al., 2004; Kessler et al., 2005). Under the influence of COVID-19, employment has produced a lot of uncertainty, and college graduates are more and more confused and anxious in job hunting and waiting (Cao, 2020). If there is a persistent intolerance of uncertainty, it will undoubtedly bring negative effects on the individual’s psychological and social adaptation. Research shows that there is a significant correlation between intolerance of uncertainty and employment anxiety of college students (Li et al., 2012).

However, career planning is the future career development plan pre-arranged by the individual system (Gould, 1979). In the transition period from school to work, career planning can improve the adaptability of individual under uncertainty (Saks and Ashforth, 2002). More importantly, career planning is negatively correlated with anxiety (Vignoli et al., 2005). Therefore, it may be important during the career transition period, especially during COVID-19.

Based on the above literature on intolerance of uncertainty, employment anxiety, and career planning, the present study addresses the following research questions:

*RQ1.* During COVID-19, is intolerance of uncertainty related to employment anxiety among graduates? And is career planning related to intolerance of uncertainty and employment anxiety?

*RQ2.* Can intolerance of uncertainty influence employment anxiety? And can career planning influence employment anxiety?

*RQ3.* Can career planning moderate the relationship between intolerance of uncertainty and employment anxiety?

The paper is structured as follows: Section “Literature Review” is devoted to the literature review. Section “Hypothesis Development” provides logical arguments for the development of the hypotheses of the study. Section “Research Methods” shows the research methods of the study, and Section “Analysis and Results” presents the analysis and results. Section “Discussion” provides a discussion. Section “Conclusion, Implications, Limitations, and Future Research Directions” presents the practical implications, limitations, and future research directions. The last section presents the conclusion of the study.

## Literature Review

### The Relationship Between Intolerance of Uncertainty and Anxiety

In all aspects of human daily life, such as physical condition, personal income, and interpersonal communication, there are many uncertainties, especially when encountering sudden events, such as natural disasters and accident disasters (Ran et al., 2018). Uncertainty is considered to be a powerful stressor and an important antecedent of anxiety (Greco and Roger, 2003; Maissi et al., 2004; Reuman et al., 2015). Experiencing the unknown due to a perceived lack of information leads to uncertainty, which undermines an individual’s ability to prepare effectively and efficiently for the future, thereby increasing the vulnerability of anxiety-related symptoms (Carleton, 2016).

Besides uncertainty, intolerance of uncertainty (IU) seems to be a significant cognitive feature of anxiety pathology (Carleton et al., 2007). IU is widely defined as cognitive, emotional, and behavioral tendencies that react negatively to uncertain situations or unpredictable future events (Freeston et al., 1994). No matter whether the outcome of the situation is positive or negative, and how likely the outcome is, this cognitive bias will make individuals tend to think that the future situation is negative and respond to the future uncertain situation in a relatively stable negative way (Buhr and Dugas, 2002). Carleton (2016) conceptualizes IU as a personality with similar traits and emphasized subjective evaluation of uncertainty. In other words, people with a high IU may find their intuitional aversion more intolerable than those with low IU. These biases may make individuals with high IU vulnerable to psychological distress (Dugas et al., 2005; Koerner and Dugas, 2008). Preliminary studies on IU have focused on its relationship with generalized anxiety disorder (GAD) and its related symptoms (e.g., Dugas et al., 1998). Specifically, increased IU can cause more anxiety and worry, which has been conceptualized as a key component of GAD (Ladouceur et al., 2000). Some people believe that IU is a risk and maintenance factor of anxiety (Shihata et al., 2017). Indeed, intolerance of future uncertainty often has an adverse effect on individuals, leading to some anxiety (Mostert and Botha, 2013).

### The Relationship Between Intolerance of Uncertainty and Employment Anxiety

Zhang (2005) believes that employment anxiety is a strong and lasting emotional experience, such as tension and anxiety, due to some problems in the process of choosing a job and concerns about the results of employment, and the corresponding physiological and behavioral changes caused by these series of concerns. Studies have shown that the uncertain event of COVID-19 has caused employment anxiety crisis of individuals (Han et al., 2020; Xu, 2020). Globally, the preliminary estimate of the International Labor Organization (Gisaor et al., 2020) indicates that about 7% of the working hours will disappear in the second half of 2020, resulting in the unemployment of up to 200 million people. Underemployment and lower wages are also likely to increase significantly, leading to an increase of 8.8 million in the number of “working poor” (Akkermans et al., 2020). These figures clearly show that COVID-19 will have a profound impact on people’s career (Akkermans et al., 2020). For a special group of college graduates, employment has produced a lot of uncertainty under the influence of COVID-19. The specific performance is that the employment market has intensified the extrusion effect on university graduates, and the employment channels are forced to shift from offline to online. The severe employment situation combined with the pressure of graduation (Huang, 2020) leads to the anxiety of nearly half of the students in job hunting and waiting (Cao, 2020). Li et al. (2012) find that college graduates’ IU is significantly positively correlated with employment anxiety. For college students who are about to graduate, the process of career change itself faces many uncertainties (Li et al., 2012). Combined with the impact of the COVID-19, the uncertainty will be exacerbated. If there is a persistent intolerance of uncertainty, it will undoubtedly have adverse effects on the individual’s psychological and social adaptation, even affect the process of career change and cause employment anxiety (Cao, 2020).

### The Meaning of Career Planning

Purpose is defined as a meaningful and relatively stable intention of an individual. It guides behavior, is characterized by active participation, and contributes to the world beyond self (Damon et al., 2003). Kohut’s (1977) self-psychology posits that a flexible and powerful ideal and goal structure is considered as a person’s direction, and plays a stabilizing role in pressure and transition period. In addition, according to the resource and perception model, purpose is also understood as a powerful psychosocial resource that can be used under pressure to help individuals suppress arousal of disgusting or challenging events (Harber et al., 2007; Burrow et al., 2015; White, 2020). The moderating role of purpose has been described convincingly. Studies have shown that higher sense of purpose is associated with lower perceived stress levels (Scheier et al., 2006). In addition, participants who were prepared to think about their purposes showed less anxiety in the real world (Burrow and Hill, 2013) and were better able to overcome obstacles and regulate emotions (McKnight and Kashdan, 2009). Purpose is the starting point and/or reference point for almost all behaviors (Dijksterhuis and Aarts, 2010), and participation in purpose-oriented actions may be a way to manage challenging emotions and actively cope with stress (McKnight and Kashdan, 2009). It may also be an important psychological resource for people of all ages to develop and/or need in this crisis, especially for young people and adults who are still developing a sense of purpose (White, 2020). As argued by Frankl (1962), “The purpose is the related structure that runs through all stages of life, especially in the environment characterized by immeasurable pressure and pain.”

Careers are structured through a planned, purpose-oriented process system (Young and Valach, 2004). Career planning refers to that a person establishes career purposes, chooses career paths, determines development plans, education plans, etc., and determines the direction, time, and program of action to achieve career purposes based on his/her own situation, current opportunities, or constraints (Gould, 1979).

### The Importance of Career Planning During COVID-19

Exploring the occupational environment for job selection is a complex task with high uncertainty, especially in the context of unstable labor market (Vignoli, 2015). In today’s social development, the pressure of employment has gradually highlighted the emotional problems of college graduates, especially after the outbreak of COVID-19, the uncertainty of the employment situation will inevitably cause graduates to breed career anxiety (Yang, 2020). Generally speaking, this kind of anxiety is higher in the group with uncertain career (Kimes and Troth, 1974; Hawkins et al., 1977). However, Saks and Ashforth (2002) found that career planning can improve adaptability of individual under uncertainty during the transition period from school to work. The career development theory of Super (1980) also points out that career planning can provide help for older teenagers and adults. More importantly, career planning was negatively correlated with anxiety (Vignoli et al., 2005). Chinese scholars believe that during COVID-19, good career planning can enable college students to make career choices and adjustments in time according to the internal and external environment, help them correctly evaluate themselves, make reasonable positioning, find the entry point to enter the society, and achieve successful employment (Liu, 2020). Therefore, career planning may be very important in uncertain environments. For college graduates, an important transitional stage of individual socialization development, it is necessary to have a certain sense of purpose and direction to help individuals resolve their emotions.

## Hypothesis Development

The employment of college graduates is not only related to personal growth and development, but also related to national economy, people’s livelihood, social harmony, and stability. However, the outbreak of COVID-19 at the beginning of 2020 has caused a certain impact on the employment of college graduates, which has resulted in a lot of uncertainty in employment (Huang, 2020). Specifically speaking, insufficient supply of jobs, and growing anxiety about employment (Wen, 2020; Yang, 2020). The process of career change itself is faced with many uncertainties, and with the impact of COVID-19, if there is a persistent intolerance of uncertainty, it will undoubtedly bring negative effects on the individual’s psychological and social adaptation, even affect the process of career change and causing employment anxiety (Cao, 2020). Previous study has proved that there is a significant positive correlation between college students’ IU and employment anxiety (Li et al., 2012). Fortunately, however, there is a significant negative correlation between career planning and anxiety (Vignoli et al., 2005), which means that career planning literature related to sense of purpose can provide wisdom on how to deal with this global adversity.

To sum up, based on previous empirical research, self-psychology, and career development theory, the present study took college graduates during COVID-19 as the research participants, analyzed the relationship between IU and employment anxiety, and further discussed the role of career planning between IU and employment anxiety. We proposed the following hypotheses:

*Hypothesis 1:* During COVID-19, there was a significant positive correlation between intolerance of uncertainty and employment anxiety, a significant negative correlation between career planning and intolerance of uncertainty, and a significant negative correlation between career planning and employment anxiety of graduates.

*Hypothesis 2:* The intolerance of uncertainty had a significant predictive effect on employment anxiety. Career planning had a significant predictive effect on employment anxiety.

*Hypothesis 3:* Career planning played a moderating role in the relationship between intolerance of uncertainty and employment anxiety.

## Research Methods

### Questionnaire Design

The present study adopted the method of questionnaire survey, which was a common method to collect a wide range of answers in empirical research. The study contained three questionnaires, which were taken from relevant literatures.

### Data Collocation

These data came from college graduates from different schools, who will face the job market within 6months to 1year. The specific process of obtaining informed consent was as follows: Firstly, we edited a “investigation guidance and feedback” through documents, including the investigation background, purpose, method, significance, and result feedback of the present study. Secondly, we send the “investigation guidance and feedback” to principals of different schools, and forward them to each class teacher after the principals agree to the investigation. Finally, under the guidance of the class teacher, each participant filled in the questionnaire on the research Web site. The author distributed 563 questionnaires through the research Web site, of which 13 were invalid, so the final valid questionnaires were 550.

### Measures

#### Intolerance of Uncertainty Scale-12

The scale was compiled by Carleton and revised by Zhang et al. (2017). Intolerance of Uncertainty Scale-12 (IUS-12) has a stable two factor structure, which represents the anxiety and avoidance components of IU. Among them, the dimension of anticipatory anxiety includes seven items, representing fear and worry about future events (e.g., “Unexpected things get on my nerves.”); the dimension of inhibitory anxiety contains five items, representing uncertainty inhibition behavior and experience (e.g., “When it comes time to take action, uncertainty holds me back.”). Using Likert 5-point method to answer, “1=completely inconsistent; 5=completely consistent.” The higher the score, the lower the level of uncertainty tolerance. In the present study, Cronbach’s *α*=0.873.

#### Vocational Selection Anxiety Questionnaire of University Graduate

This questionnaire was revised by Zhang and Yao (2011). It has 26 items and is divided into four dimensions, employment competition pressure (e.g., “A failed job application by a nearby classmate has made me more worried about employment.”), lack of employment support (e.g., “I don’t know how to prepare employment materials.”), lack of self-confidence (e.g., “I am anxious that my academic performance is not outstanding enough.”), and worry about employment prospects (e.g., “I am worried that my career ideals will not be realized.”). Using Likert 5-point method to answer, “1=completely inconsistent; 5=completely consistent.” The higher the score, the higher the employment anxiety. In the present study, Cronbach’s *α*=0.967.

#### Career Planning Scale

Adopting the Career Planning Scale compiled by Lin (1992, Unpublished) according to Osipow et al. (1976) and revised by Chen (1987). It includes four dimensions: self-awareness (e.g., “I still don’t know much about my interests.”), self-confidence (e.g., “I don’t have much confidence in myself.”), career purpose establishment (e.g., “I can’t set a specific career goal right now.”), and career decision-making ability (e.g., “There are so many majors and careers that appeal to me, I don’t know which one to prioritize.”). A total of 26 questions were answered by Likert 5-point method, with reverse scoring, “5=very consistent; 1=very inconsistent.” The higher the score, the lower the level of career planning. In the present study, the Cronbach’s *α*=0.971.

### Demographics

Most of the participants were female (24.5% male; 75.5% female); 37.1% of the participants were science and engineering majors, 30.0% were humanities and social sciences majors, 9.6% were management majors, 10.4% were art majors, and 12.9% were other majors; and 21years old accounted for 1.3%, 22years old accounted for 45.8%, 23years old accounted for 38.5%, 24years old accounted for 10.5%, 25years old accounted for 2.9%, and 26years old accounted for 0.9%. Table 1 showed the demographic data of the participants.

**Table 1:** Demographics.

Characteristics	Category	Frequency (*n*)	Percentage (%)
Gender	Male	135	24.5
	Female	415	75.5
Age	21	7	1.3
	22	252	45.8
	23	212	38.5
	24	58	10.5
	25	16	2.9
	26	5	0.9
Major	Science & Engineering	204	37.1
	Humanities & Social Sciences	165	30.0
	Management	53	9.6
	Art	57	10.4
	Other	71	12.9

## Analysis and Results

### Descriptive Statistics

SPSS 21.0 was used for data analysis. The descriptive statistical results of each questionnaire score were shown in Table 2.

**Table 2:** The descriptive statistical results of each scale.

	*M*	*SD*	*N*
Anticipatory anxiety	2.676	0.642	550
Inhibitory anxiety	2.586	0.632	550
IU	5.262	1.204	550
Employment competition pressure	2.435	0.774	550
Lack of employment support	2.763	0.641	550
Lack of self-confidence	2.794	0.397	550
Worry about employment prospects	2.716	0.555	550
Employment anxiety	10.708	2.063	550
Self-awareness	3.394	0.776	550
Self-confidence	3.422	0.925	550
Career purpose establishment	3.460	0.966	550
Career decision-making ability	3.602	0.913	550
Career planning	13.878	3.277	550

### Correlation Analysis

The results of correlation analysis (Table 3) showed that there was a significant positive correlation between IU and employment anxiety, a significant negative correlation between career planning and IU, and a significant negative correlation between career planning and employment anxiety. The result was consistent with hypothesis (1).

**Table 3:** Descriptive statistics and correlation coefficient of each variable.

	1	2	3	4	5	6	7	8	9	10	11	12	13
Anticipatory anxiety	1												
Inhibitory anxiety	0.787^**^	1											
IU	0.946^**^	0.944^**^	1										
Employment competition pressure	0.544^**^	0.555^**^	0.581^**^	1									
Lack of employment support	0.551^**^	0.525^**^	0.570^**^	0.771^**^	1								
Lack of self-confidence	0.501^**^	0.492^**^	0.525^**^	0.708^**^	0.660^**^	1							
Worry about employment prospects	0.519^**^	0.526^**^	0.553^**^	0.644^**^	0.610^**^	0.577^**^	1						
Employment anxiety	0.612^**^	0.608^**^	0.645^**^	0.924^**^	0.891^**^	0.819^**^	0.811^**^	1					
Self-awareness	−0.503^**^	−0.480^**^	−0.520^**^	−0.690^**^	−0.701^**^	−0.575^**^	−0.606^**^	−0.750^**^	1				
Self-confidence	−0.595^**^	−0.561^**^	−0.611^**^	−0.716^**^	−0.669^**^	−0.633^**^	−0.602^**^	−0.761^**^	0.723^**^	1			
Career purpose establishment	−0.509^**^	−0.477^**^	−0.522^**^	−0.675^**^	−0.675^**^	−0.579^**^	−0.582^**^	−0.731^**^	0.744^**^	0.855^**^	1		
Career decision-making ability	−0.514^**^	−0.512^**^	−0.543^**^	−0.687^**^	−0.656^**^	−0.575^**^	−0.571^**^	−0.726^**^	0.719^**^	0.790^**^	0.837^**^	1	
Career planning	−0.581^**^	−0.555^**^	−0.601^**^	−0.756^**^	−0.737^**^	−0.646^**^	−0.644^**^	−0.819^**^	0.861^**^	0.926^**^	0.946^**^	0.919^**^	1

* *p < 0.05;*

** *p < 0.01; and*

*** *p < 0.001.*

### Regression Analysis

SPSS 21.0 and Interaction 1.7 were used to test the moderating effect of career planning on the relationship between IU and employment anxiety. Taking IU, career planning and IU×career planning as independent variables and employment anxiety as dependent variables. In order to avoid the negative effects of multicollinearity among variables, we have made a central treatment of IU and career planning (Aiken and West, 1991). Gender as a covariate was included in the regression equation; however, its coefficient was not significant, so it was eliminated. The first step was IU and career planning after centralization; the second step was IU×career planning after centralization.

According to the results of hierarchical multiple regression analysis (Table 4), the prediction effect of IU on employment anxiety was significant, *B*=0.424, 95% CI (0.325, 0.523), *SE*=0.051, *p*<0.001. Career planning had a significant predictive effect on employment anxiety, *B*=−0.416, 95% CI (−0.453, −0.380), *SE*=0.019, *p*<0.001. The result was consistent with hypothesis (2). The interaction of IU×career planning had a significant predictive effect on employment anxiety, *B*=0.028, 95% CI (0.008, 0.048), *SE*=0.010, *p*<0.01. The results showed that career planning had a moderating role on the relationship between IU and employment anxiety. This result preliminarily verified hypothesis (3).

**Table 4:** The results of multiple regression analysis of adjustment model (*n*=550).

Step	Result variable	Predictive variable	*B*	*SE*	*t*	*p*	*R* ^2^	*R*^2^ change	*F* change
1	Employment anxiety	IU	0.424	0.051	8.393	0.000^***^	0.696	0.696	625.410^***^
		Career planning	−0.416	0.019	−22.424	0.000^***^			
2	Employment anxiety	IU	0.411	0.050	8.137	0.000^***^			
		Career planning	−0.415	0.018	−22.456	0.000^***^	0.700	0.004	7.337^**^
		IU×career planning	0.028	0.010	2.709	0.007^**^			

^*^ *p<0.05;*

** *p<0.01; and*

*** *p<0.001*.

**Figure 1:**
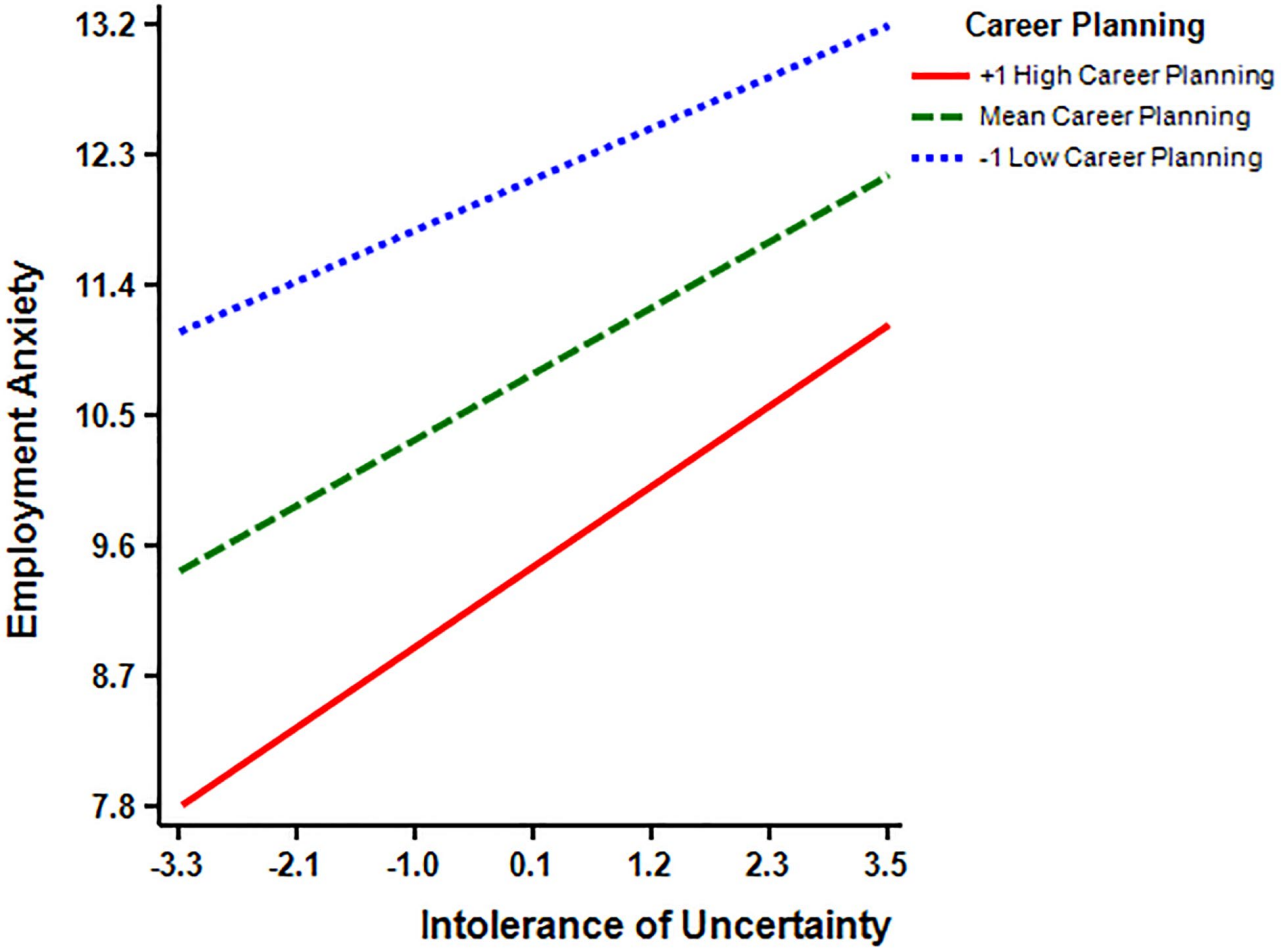
Simple slope test.

In order to further test the moderating role of career planning, it was divided into high and low groups by adding and subtracting a standard deviation from the average, and the influence of IU on employment anxiety at different levels of career planning was investigated (Simple Slope Test). The result showed that the impact of IU on employment anxiety increased with the increase of career planning. The impact of IU on employment anxiety was significant at all levels of career planning, but at the lowest level of career planning (M−1 SD; simple slope=0.318, *t*=5.006, *p*<0.001). It was the weakest in the middle level of career planning (M; simple slope=0.410, *t*=8.121, *p*<0.001), and the strongest at the highest level of career planning (M+1 SD; simple slope=0.502, *t*=8.673, *p*<0.001; Figure 1).

## Discussion

The present study investigated whether self-reported career planning could moderate the relationship between intolerance of uncertainty and employment anxiety of graduates during COVID-19. The results of the present study supported to hypothesis (1) that there was a significant positive correlation between IU and employment anxiety. This result was consistent with previous conclusions. Li et al. (2012) found that intolerance of uncertainty was significantly positively correlated with employment anxiety. In addition, the present study found that there was a significant negative correlation between career planning and employment anxiety, as well as the relationship between career planning and IU. This conclusion was supported by previous-related research results. Career planning was negatively correlated with anxiety (Vignoli et al., 2005), which can improve people’s adaptability under uncertainty (Saks and Ashforth, 2002).

The result of regression analysis was supported to hypothesis (2) that IU can significantly positively predict employment anxiety, that was, the higher IU, the stronger employment anxiety. Those who cannot tolerate uncertainty may think that they lack the necessary response resources and problem-solving ability to effectively deal with threatening situations, which stimulates their physical discomfort and negative emotions and reduces their willingness to take action to make decisions and solve problems (Buhr and Dugas, 2002). In other words, college students who cannot tolerate uncertainty will show anxiety tendency when choosing a career because they cannot cope effectively without knowing the exact result. This also showed that anxiety was a common emotional experience in the face of uncertain situations (Shihata et al., 2017).

Moreover, the findings of the present study supported hypothesis (3) that career planning played a moderating role between IU and employment anxiety. Specifically, the higher the ability of career planning, college graduates would significantly reduce their employment anxiety in the face of uncertain events or situations. This finding showed that college graduates with employment anxiety could benefit from career planning, that was, despite the severe stress events, but with the support of a sense of purpose, their anxiety would be reduced. The results of the present study were similar to the findings of other stressors related to sense of purpose in western literature. Preexisting purposes can help buffer the effects of stress and promote emotional recovery in adversity (McKnight and Kashdan, 2009; Burrow and Hill, 2013).

## Conclusion, Implications, Limitations, and Future Research Directions

### Conclusion

The outbreak of COVID-19 has disrupted many aspects of our lives. In the face of the severe epidemic situation, it has become extremely difficult for graduates to obtain employment. Our research showed that in the context of COVID-19, intolerance of uncertainty of graduates was significantly positively correlated with employment anxiety, and the effective way to deal with employment anxiety was to pay attention to career planning, because career planning can significantly moderate the relationship between intolerance of uncertainty and employment anxiety. Our results were consistent with self-psychology and career planning theory, and demonstrate that career planning related to sense of purpose can improve individuals’ adaptability under uncertain situations.

### Implications

During COVID-19, the lower the tolerance for uncertainty among college graduates, the higher their employment anxiety. The reason is that people with high IU believe that they lack the necessary coping resources and problem-solving ability to effectively deal with threatening situations, which stimulates their physiological discomfort and negative emotions, and reduces their willingness to take actions to make decisions and solve problems. In other words, without knowing the exact result, we cannot deal with it effectively, which directly leads to college students with high IU showing excessive anxiety tendency in the process of choosing a job. This also shows that anxiety is a common emotional experience in the face of uncertain situations.

Career planning has a moderating effect on the relationship between IU and employment anxiety. This finding suggests that career planning can improve the adaptability of college graduates under uncertain situations, and significantly reduce their employment anxiety during the pandemic. College graduates with high employment anxiety can benefit from career planning, that is, although facing more serious stressful events, their anxiety will be reduced if they are supported by a sense of purpose. Research has shown that preexisting purpose can help buffer the influence of stress and promote emotional recovery during adversity.

To sum up, the present study provides a certain theoretical basis. To be specificity, is very important to guide college students to determine their career goals and pay attention to the cultivation of students’ career planning ability. Schools should set up professional departments to take full-time responsibility for the vocational education of all students and combine professional guidance with basic guidance to carry out vocational education in an all-round way. At the same time, the professional career assessment scale is introduced to provide scientific reference for the determination of students’ career goals, which will help graduates to actively cope with COVID-19, even the uncertainty situation in the future, and help schools put forward appropriate strategies to actively respond to this global adversity.

### Limitations and Future Research

The present study also had some limitations and deficiencies. First of all, in theory, although the research had successfully proved the moderating role of career planning on IU and employment anxiety, the participants were not representative enough. According to career development stage theory of Super, everyone’s career stage should match with career development. All the participants selected in our study were college graduates. We do not know whether the sense of purpose could also help other adults cope with negative emotions when facing the uncertain situation of career development, which needs to be verified in the future. Secondly, the present study has not manipulated IU levels for decreasing, therefore, how to weaken the influence of uncertainty caused by COVID-19 on employment anxiety and help the graduates to cope with negative emotions better is particularly important. Lastly, the growth or consolidation of purpose awareness can be used as an important internal resource in times of adversity (White, 2020). Many experiments provide convincing evidence that people usually find or impose meaning and coherence when they are uncertain (Heine et al., 2006; Sedikides and Wildschut, 2018). However, studies have shown that loss of income, concerns about the labor market, and limited work experience and social opportunities may impair the sense of purpose (Clark et al., 2010). Therefore, how to keep or feel the purpose and realize the importance of career planning is a problem worthy of consideration. We look forward to continuing to focus on the interaction between people and the environment and to discover lasting ways to help people maintain a sense of purpose.

## Data Availability Statement

The raw data supporting the conclusions of this article will be made available by the authors, without undue reservation.

## Ethics Statement

The studies involving human participants were reviewed and approved by the scientific and research Ethics Committee of the School of Psychology, NWNU. The patients/participants provided their written informed consent to participate in this study.

## Author Contributions

LC was responsible for the implementation of the collection and analysis of the data. SZ was mainly responsible for the conception and design of the writing of articles. All authors contributed to the article and approved the submitted version.

## Funding

This work was supported by Northwest Normal University (grant numbers 2018SKGG06 and NWNU-LKQN-18-36).

## Conflict of Interest

The authors declare that the research was conducted in the absence of any commercial or financial relationships that could be construed as a potential conflict of interest.

## Publisher’s Note

All claims expressed in this article are solely those of the authors and do not necessarily represent those of their affiliated organizations, or those of the publisher, the editors and the reviewers. Any product that may be evaluated in this article, or claim that may be made by its manufacturer, is not guaranteed or endorsed by the publisher.
